# Supplementary material for: Input Connectivity Reveals Additional Heterogeneity of Dopaminergic Reinforcement in Drosophila
Source: Curr Biol. 2020 Aug 17;30(16):3200–3211.e8. doi: 10.1016/j.cub.2020.05.077 (PMC7443709; doi:10.1016/j.cub.2020.05.077)
Supplement: Data S1. Nervous System Expression Patterns for Key GAL4 Driver Lines, Related to Figures 3 and 4 and STAR Methods [file mmc6.pdf]

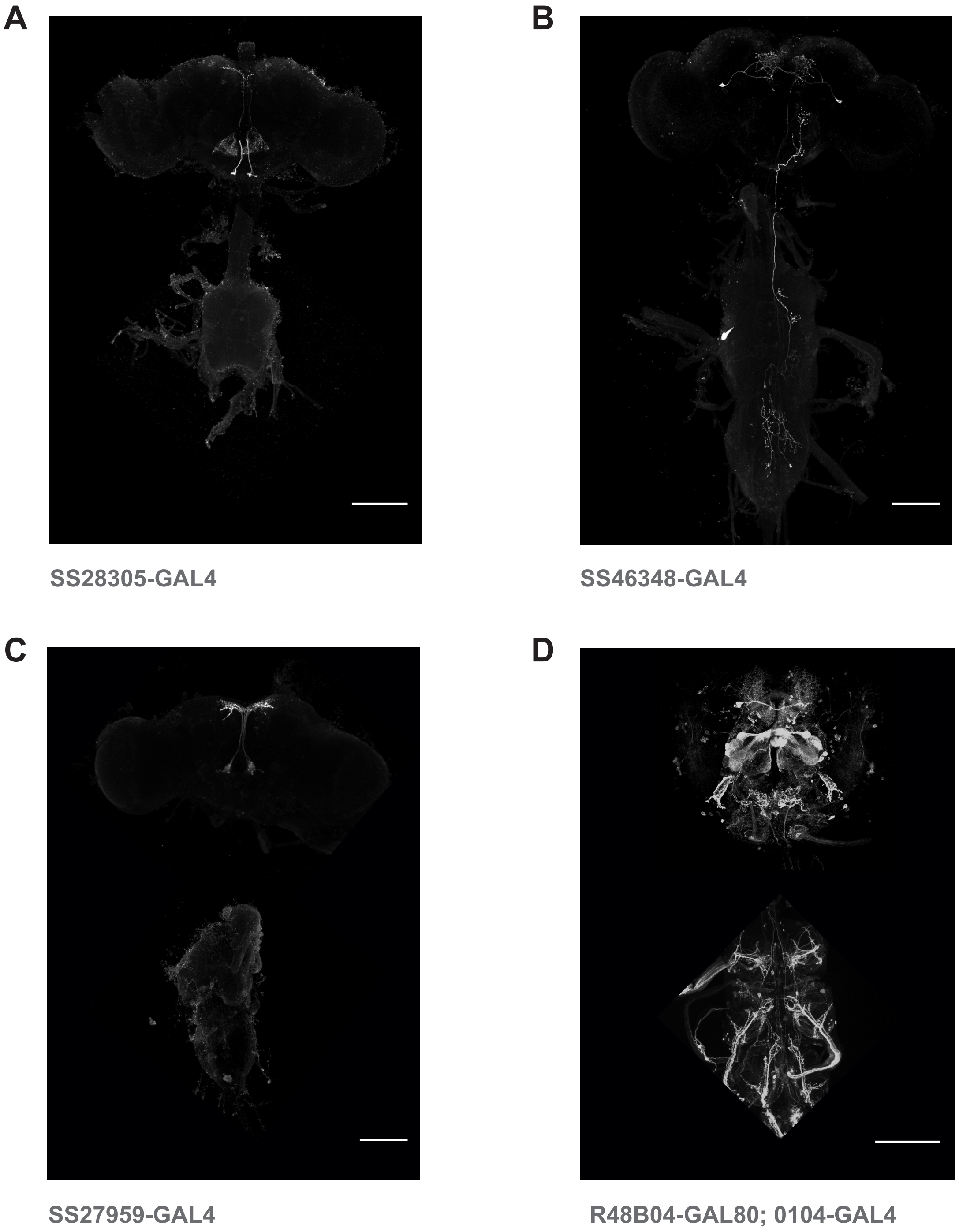

Maximum intensity projections of confocal imaging stacks of Drosophila central and ventral nervous systems (CNS and VNC). Key GAL4 driver flies used in the functional experiments are shown expressing GFP (grey) in neurons of interest. Scalebar, 100μm. Due to COVID-19 enforced restrictions, we direct readers to the below listed publicly accessible databases for details/images of the other lines used in this study.

Name	Collection	CNS and VNC
GMR58E02-GAL4	FlyLight	For FlyLight and Vienna Tile lines:
GMR88F08	FlyLight	https://www.janelia.org/project-team/flylight/tools-and-reagents
GMR66C08	FlyLight	
MB320C-GAL4	FlyLight	
MB315C-GAL4	FlyLight	
VT006202-GAL4	Vienna Tiles	For the InSite Collection:
0104-GAL4	InSite	http://www.columbia.edu/cu/insitedatabase/
0804-GAL4	InSite	(W. Grueber and T. Clandinin Labs)
