# Supplementary material for: Input Connectivity Reveals Additional Heterogeneity of Dopaminergic Reinforcement in Drosophila
Source: Curr Biol. 2020 Aug 17;30(16):3200–3211.e8. doi: 10.1016/j.cub.2020.05.077 (PMC7443709; doi:10.1016/j.cub.2020.05.077)

Revision Status Table

Table details the review status of each DAN traced and analysed in this study. The total number of identified dendritic postsynapses on each DAN and the percentage of the postsynapses that are connected to an input neuron that was traced to identification are listed. Only DANs that were expert reviewed and have more than 50 identified input neurons were included in the analyses.

Publication name	CATMAID Skeleton ID	Review status	Total dendritic post synapses	% of dendritic post synapses connected to input neuron	Included for analyses (total (inputs IDed) >50)
γ5-1(fb)	3639761	standard expert review<br>+ extensive review of the dendrite	288	94.1	✓
γ5-5(fb)	3643424	standard expert review<br>+ extensive review of the dendrite	183	91.3	✓
γ5-6(fb)	5234721	standard expert review<br>+ extensive review of the dendrite	217	91.2	✓
γ5-19(uc)	6181246	standard expert review<br>+ extensive review of the dendrite	204	91.2	✓
γ5-20(uc)	3134936	standard expert review<br>+ extensive review of the dendrite	322	89.4	✓
γ5-23(uc)	11262395	standard expert review<br>+ extensive review of the dendrite	186	88.2	✓
γ5-24(uc)	11274761	standard expert review	148	82	✓
γ5-25(uc)	11149656	standard expert review (4%)	137	90	✓
γ5-16(lc)	6019056	standard expert review	268	45	✓
γ5-10(lc)	5652208	standard expert review	247	81	✓
γ5-12(lc)	5914197	standard expert review	225	52	✓
γ5-2(lc)	3025479	standard expert review	123	87	✓
γ5-7(lc)	3026119	standard expert review	106	96	✓
γ5-4(lc)	3143746	standard expert review	95	58	✓
γ5-13(lc)	5952626	standard expert review	72	97	✓
γ5-21(da)	11169413	standard expert review (60%)	109	77	✓
γ5-15(ba)	6013704	standard expert review	145	93	✓
γ5-17(ba)	6059582	standard expert review<br>+ extensive review of the dendrite	239	94.1	✓
β'2a-1(2)	7777207	standard expert review<br>+ extensive review of the dendrite	307	92.5	✓
β'2a-2(nc)	7270967	standard expert review<br>+ extensive review of the dendrite	169	91.1	✓
β'2a-4(1)	8317886	standard expert review	281	93	✓

β'2a-3(2)	7512499	standard expert review (30%)	212	45	✓
PPL1-γ1pedc	1159799	standard expert review<br>+ extensive review of the dendrite	3480	46.9	✓
**γ5-22**	11175371	standard expert review	94	46	
**γ5-8**	5631471	standard expert review (73%)	44	59	
**β'2a-5**	6969526	not reviewed (not fully reconstructed)	3	33	
**β'2a-6**	5886218	not reviewed (not fully reconstructed)	4	75	

A multistep clustering approach to classify DAN input neurons
(Left) Coarse clusters are generated by hierarchical clustering of an ‘all-by-all’ NBLAST comparison of simplified neurons. Neurons are simplified leaving the soma tract and the main neurite. This first step works unsupervised on all 821 neurons, resulting in 20 coarse clusters (Figure 3I). Middle: Larger primary clusters are subclustered by weighting defining neural features. 80% towards the core neurons and 20% towards the complement of the core neuron - “the fine branches” (green). This example shows SEZONs which are comprised of long tracts and a simple branched arbor. This created 153 sub-clusters with little human supervision. (Right) Fine clustering. Neurons in each sub cluster have different key morphological features thus subsequent clustering steps are different in exact parameters. These settings are reviewed manually. We determined the step - fine clustering – in most cases to be sufficiently accurate to separate all neurons that are morphologically different in different fine clusters.

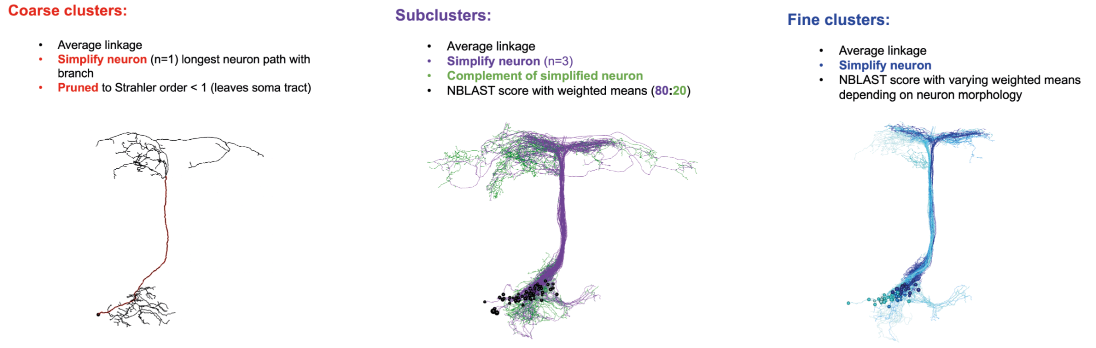

Mantel test to determine statistical significance of correlations between properties (cluster results) compared in tanglegrams

Here the morphological NBLAST distance matrix is compared to the observed connectivity distance matrix in Figure 2E. Comparison is by Pearson's correlation of the Manhattan distributions. The Connectivity matrix is subsequently shuffled 10^7 times and the resulting matrices are compared to the observed NBLAST distance matrix. Lastly (right), the correlation coefficients that are higher than the correlation coefficient between the observed data (morphology and connectivity) are divided by the number of randomisations (10^7) to obtain a p-value. A small p-value suggests the compared distance matrices are correlated.

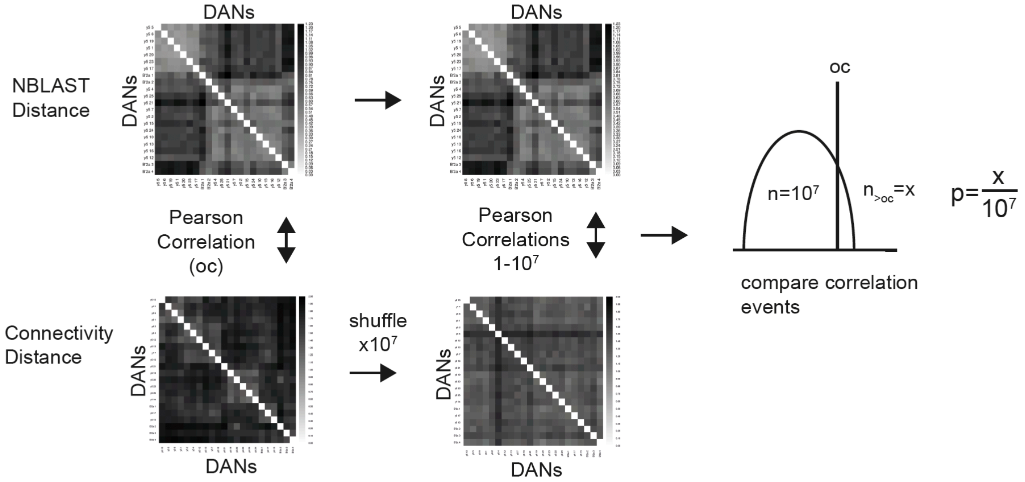

Random connectivity null model

CATMAID synaptic connectivity from observed data is represented as a connectivity matrix (left). This matrix is randomised conserving the proportion of DAN postsynaptic budget and input presynaptic budget in the network. 10^4 random matrices are computed for both the observed connectivity matrix and the random matrix, normalised by DAN postsynaptic budget (middle). A Manhattan distances between observed and simulated matrices are calculated. (B) Statistical analysis of mean Manhattan distances is carried out by comparing the distribution of Manhattan distances in the observed data (red) with the mean distribution of 10^4 simulated matrices (blue). The means of the two distributions are significantly different; $p<0.0001$. Therefore, the organization of DAN input connectivity is not random.

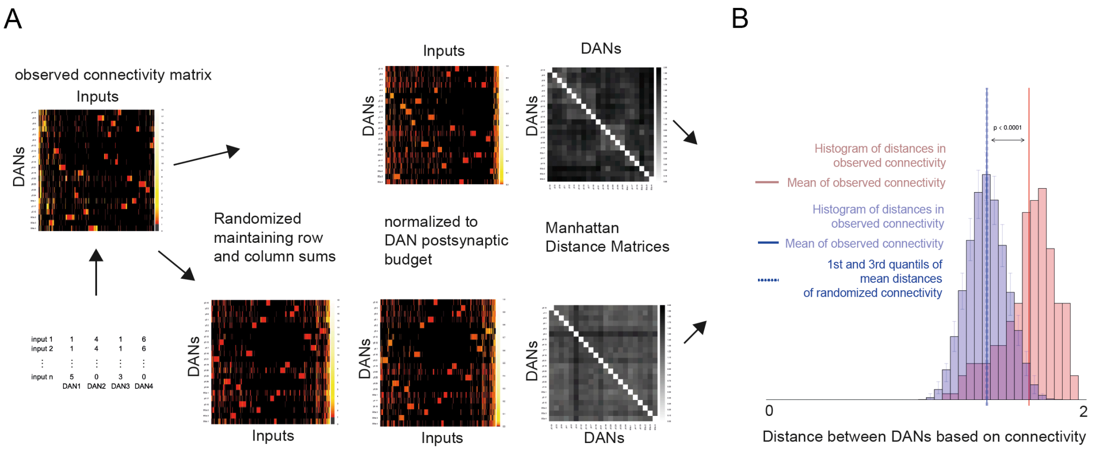

Supplement: Methods S1. Revision Status Table and Pictographical Explanations of Neuron Clustering Strategy and Statistical Testing, Related to STAR Methods [file mmc7.pdf]
